# Anomalous Origin of the Left Circumflex Coronary Artery: Approach in Acute Coronary Syndrome

**DOI:** 10.7759/cureus.29330

**Published:** 2022-09-19

**Authors:** Pradnya Brijmohan Bhattad, Eddison Ramsaran

**Affiliations:** 1 Cardiovascular Medicine, Saint Vincent Hospital, University of Massachusetts Chan Medical School, Worcester, USA

**Keywords:** st-elevation myocardial infarction, cocaine-induced coronary disease, acute stent thrombosis, acute coronary syndrome, coronary artery disease, anomalous origin of left circumflex artery

## Abstract

Coronary artery anomalies are congenital and relatively uncommon. Anomalous origin of the left circumflex artery from the right is a relatively common congenital coronary variant and is usually considered benign in itself. The presence of an anomalous coronary artery may pose challenges in engaging the anomalous vessel, and prompt recognition of an anomalous coronary artery is important to allow for appropriate coronary interventions.

Here, we describe the case of a patient who presented with cocaine-induced acute ST-segment elevation myocardial infarction and was incidentally noted to have an anomalous left circumflex coronary artery arising from the right coronary cusp with a relatively uncommon variant of the anomalous origin. We believe that this case in itself is rare and discusses the approach to anomalous coronaries in an acute coronary syndrome presentation which is unique and rare in the existing literature regarding coronary anomalies.

## Introduction

Coronary artery anomalies are rare and may be seen in patients without any evidence of structural heart disease. Anomalous left circumflex coronary artery (LCx) originating from the right coronary artery (RCA) or from the right sinus of Valsalva is one of the common congenital coronary anomalies seen in patients who undergo coronary angiography [1].

Anomalous origin of the LCx from the RCA is usually considered a benign finding and does not lead to any hemodynamic effect. The anomalous LCx course is almost always posterior to the aorta and is considered a benign coronary anomaly [2].

## Case presentation

**Figure 1:**
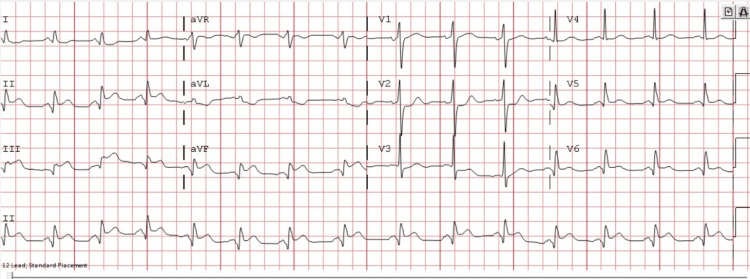
Electrocardiogram showing sinus rhythm with ST-segment elevation in leads II, III, aVF, and ST-segment depressions in leads V2-V4.

A 58-year-old male with a history of cocaine use and daily cigarette smoking for the past several years with no known previous medical history presented to the emergency room (ER) with sudden onset of severe substernal chest pain radiating down his left arm with associated nausea and diaphoresis. He reported using cocaine a day prior to his presentation. His blood pressure was 148/93 mmHg, heart rate was in the 90s beats/minute, oxygen saturation of 95% on room air, and an unremarkable physical examination at the time of presentation. An electrocardiogram (ECG) showed ST-segment elevation in the inferior leads (Figure 1).

The cardiac catheterization laboratory was immediately activated for ST-elevation myocardial infarction (STEMI). His complete blood cell count and a chemistry panel were within normal limits. His initial cardiac troponin T was negative/less than 0.030 ng/dL. He received a loading dose of aspirin, ticagrelor, statin therapy, and a bolus of heparin and was taken for emergent coronary angiography. Moreover, 100% proximal stenosis of the RCA was noted on the coronary angiogram for which he underwent revascularization with a single drug-eluting stent to the RCA (Video 1). LCx had an anomalous origin from the right coronary cusp as noted on the coronary angiogram (Videos 1-5).

**Video 1:** Anomalous LCx originating from the right coronary cusp is visualized here. Occluded proximal RCA is noted from the first STEMI presentation. LCx: left circumflex coronary artery; RCA: right coronary artery; STEMI: ST-elevation myocardial infarction

**Video 2:** RCA after revascularization with a drug-eluting stent. Anomalous LCx originating from the right coronary cusp is visualized here. RCA: right coronary artery; LCx: left circumflex coronary artery

**Video 3:** Coronary angiogram of the left system but no LCx visualized here in the same patient. LCx: left circumflex coronary artery

**Video 4:** Coronary angiogram with missing LCx origin here on engaging the left main. LCx: left circumflex coronary artery

**Video 5:** LCx origin is missing here on a coronary angiogram with the catheter engaging the left main and only the left anterior descending artery is seen from the left main. LCx: left circumflex coronary artery

**Figure 2:**
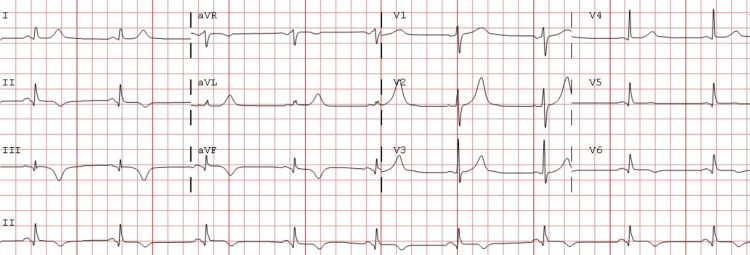
Electrocardiogram from the same patient showing sinus rhythm with T-wave inversions in the inferior leads with the resolution of ST-segment elevation in the same leads.

A transthoracic echocardiogram revealed a left ventricular ejection fraction of 55% with moderate inferior and posterior wall hypokinesis. Post-revascularization, he was noted to have a peak of troponin T at 7 ng/dL followed by a fall in cardiac troponin T levels. Post-revascularization, his ECG changes of ST-segment elevation resolved (Figure 2).

He was discharged home on guideline-directed medical therapy with dual antiplatelet therapy, beta-blocker, and a statin.

**Figure 3:**
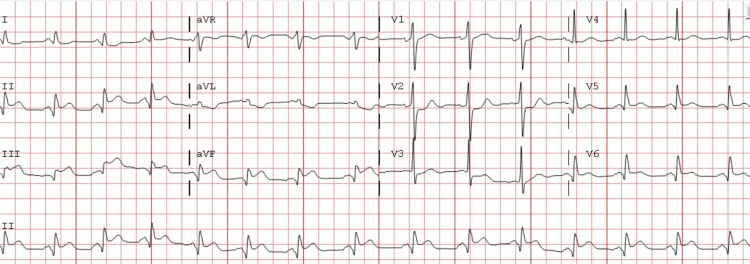
Electrocardiogram in the same patient five days after the initial STEMI presentation showing new ST-segment elevation in the inferior leads with ST depression in V2-V3 leads. STEMI: ST-elevation myocardial infarction

The patient presented again to the ER five days after the initial discharge with sudden onset of severe substernal chest pain radiating down his left arm, and an ECG revealed inferior ST-segment elevations with ST depressions in the leads V2-V3 (Figure 3).

He reported not taking any of his medications including dual antiplatelet therapy that he was prescribed at discharge five days earlier. Again cardiac catheterization laboratory was emergently activated and he underwent an emergent coronary angiogram after receiving a bolus of heparin, aspirin, and ticagrelor therapy. A repeat emergent coronary angiogram revealed a stent thrombus in the RCA for which he underwent an aspiration thrombectomy and balloon angioplasty (Videos 6, 7).

**Video 6:** Coronary angiogram after the second STEMI presentation demonstrating RCA thrombosis and anomalous LCx originating from the right sinus. STEMI: ST-elevation myocardial infarction; RCA: right coronary artery; LCx: left circumflex coronary artery

**Video 7:** RCA after aspiration thrombectomy and balloon angioplasty after the second STEMI presentation in the same patient, anomalous LCx is well visualized here. RCA: right coronary artery; STEMI: ST-elevation myocardial infarction; LCx: left circumflex coronary artery

**Figure 4:**
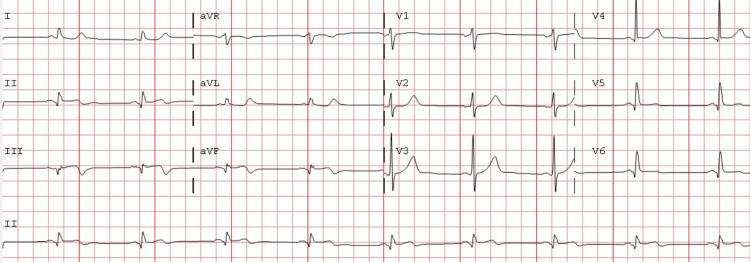
Electrocardiogram in the same patient post-aspiration thrombectomy after the second presentation showing resolving ST-segment elevation with T-wave inversion in the inferior leads.

He was started on eptifibatide and bivalirudin continuous infusions for eight to twelve hours post-thrombectomy after which these agents were discontinued. Post-thrombectomy, he was noted to have an improvement in his symptoms, the resolution of ST-segment elevation on ECG (Figure 4) with a downward trend of cardiac biomarkers.

He was counseled on strict cessation of cocaine and was advised strict medication compliance. He was subsequently discharged on guideline-directed medical management with dual antiplatelet therapy, beta-blocker, angiotensin-converting enzyme inhibitor, and statin therapy. He will be followed as an outpatient.

## Discussion

Anomalous LCx originating from the right sinus or RCA usually always courses retroaortic [2]. As such, this coronary anomaly does not carry the risk of compression between the aorta and pulmonary artery, especially with stress or exertion, and, as such, has been considered a relatively benign entity. Other congenital coronary anomalies have been described to have an interarterial course which is considered a malignant course for an anomalous coronary vessel due to its potential to be compressed between the aorta and pulmonary artery and exercise-induced ischemia with a high risk for sudden cardiac death. However, this risk has not been seen in cases of an anomalous LCx from the right because of its relatively benign course [1,3].

An anomalous LCx may be occasionally difficult to engage on coronary angiogram making it challenging during coronary interventions, especially in the setting of acute coronary syndrome presentations. There may be several variants of an anomalous origin of LCx from the right wherein there may be common ostia for both the RCA and LCx in the right sinus of Valsalva or there may be separate ostia. Anomalous LCx originating as a branch of the proximal RCA has been described as well [1,3,4].

Usually, an anomalous LCx is an incidental finding but need to be vigilant to promptly recognize the anomalous origin of LCx given that it is a relatively uncommon entity and may pose challenges to engage and recognize it angiographically. Inadequate visualization and recognition of an anomalous LCx may pose challenges during revascularization [2,4,5]. This is especially important in acute coronary syndrome where the door-to-balloon time is very crucial in timely management.

When a first left angiographic view on coronary angiogram shows an unusually long left main artery, an anomalous origin of LCx should be suspected. The unusually long left main on the first left angiographic view would be likely the left anterior descending artery given the fact that LCx is missing from the angiographic view of the left system.

The presence of an anomalous LCx in the setting of acute coronary syndrome presentation may make a challenging scenario for coronary interventions if not recognized appropriately and promptly.

The important reasons to evaluate complete coronary anatomy prior to percutaneous coronary intervention (PCI) of a suspected lesion in a STEMI are summarized as follows: (1) The presence of an anomalous coronary artery may be identified which may be the true culprit lesion. (2) Eliminates the time utilized looking for a suspected missing vessel when in fact it has an anomalous origin. (3) Information about the duration and characteristics of an occluded vessel may be provided by retrograde collaterals/filling. (4) The state of the non-culprit vessels may aid in the level of hemodynamic support needed for proceeding to PCI.

## Conclusions

Our case adds rare aspects in the evaluation of anomalous LCx in the setting of a STEMI where the door-to-balloon time is very crucial for management. The overall prevalence of coronary anomalies is <2% in the general population, and, as such, these anomalies are rarely encountered in regular clinical settings. Even rarer is the presentation of an anomalous coronary in an acute coronary syndrome situation with challenges in prompt revascularization due to anomalous coronary anatomy.
